# Implementation and feasibility of the stroke nursing guideline in the care of patients with stroke: a mixed methods study

**DOI:** 10.1186/s12912-017-0262-y

**Published:** 2017-12-01

**Authors:** Ingibjörg Bjartmarz, Helga Jónsdóttir, Thóra B. Hafsteinsdóttir

**Affiliations:** 1 Clinical Nurse Specialist, Department of Rehabilitation, Landspítali University Hospital, Reykjavík, Iceland; 2 Faculty of Nursing, University of Iceland, Reykjavík, Iceland; 3 Nursing Care for Chronically Ill Adults, Landspítali University Hospital, Reykjavík, Iceland; 4 Julius Center for Health Sciences and Primary Care, Nursing Science Department, University Medical Center Utrecht, Utrecht, The Netherlands

**Keywords:** Stroke, Nursing, Evidence based care, Clinical practice guidelines, Feasibility studies

## Abstract

**Background:**

Nurses often have difficulties with using interdisciplinary stroke guidelines for patients with stroke as they do not focus sufficiently on nursing. Therefore, the Stroke Nursing Guideline (SNG) was developed and implemented. The aim of this study was to determine the implementation and feasibility of the SNG in terms of changes in documentation and use of the guideline in the care of stroke patients on Neurological and Rehabilitation wards, barriers and facilitators, and nurses’ and auxiliary nurses’ view of the implementation.

**Methods:**

A sequential explorative mixed method design was used including pre-test post-test measures and post intervention focus groups interviews. For the quantitative part retrospective electronic record data of nursing care was collected from 78 patients and prospective measures with Barriers and Facilitators Assessment Instrument (BFAI) and Quality Indicator Tool (QIT) from 33 nursing staff including nurses and auxiliary nurses. In the qualitative part focus groups interviews were conducted with nursing staff on usefulness of the SNG and experiences with implementation.

**Results:**

Improved nursing documentation was found for 23 items (*N* = 37), which was significant for nine items focusing mobility (*p* = 0.002, *p* = 0.024, *p* = 0.012), pain (*p* = 0.012), patient teaching (*p* = 0.001, *p* = 0.000) and discharge planning (*p* = 0.000, *p* = 0.002, *p* = 0.004). Improved guideline use was found for 20 QIT-items (*N* = 30), with significant improvement on six items focusing on mobility (*p* = 0.023), depression (*p* = 0.033, *p* = 0.025, *p* = 0.046, *p* = 0.046), discharge planning (*p* = 0.012). Facilitating characteristics for change were significantly less for two of four BFAI-subscales, namely Innovation (*p* = 0.019) and Context (*p* = 0.001), whereas no change was found for Professional and Patient subscales. The findings of the focus group interviews showed the SNG to be useful, improving and providing consistency in care. The implementation process was found to be successful as essential components of nursing rehabilitation were defined and integrated into daily care.

**Conclusion:**

Nursing staff found the SNG feasible and implementation successful. The SNG improved nursing care, with increased consistency and more rigorous functional exercises than before. The SNG provides nurses and auxiliary nurses with an important means for evidence based care for patients with stroke. Several challenges of implementing this complex nursing intervention surfaced which mandates ongoing attention.

**Electronic supplementary material:**

The online version of this article (10.1186/s12912-017-0262-y) contains supplementary material, which is available to authorized users.

## Background

Stroke generally results in life-altering changes for both patients and their closest family. Patients experience a whole arena of physical and psychosocial impairments [1]. In the long term 25–74% of patients have to rely on assistance of family for the help in basic Activities of Daily Living (ADL’s) like feeding, self-care, and mobility due to the physical impairments, like paralysis of one side of the body, decrease in abilities such as reaching and handling objects [2]. Difficulties with posture and balance make it difficult for patients to walk and mobilize. About one-third of patients are confronted with cognitive impairments such as speaking and comprehending language [3] and many patients have difficulties with memory, which makes it difficult for patients to acquire and maintain new information [4]. Patients are confronted with the huge challenges due to changes in self-identity, role capacity and their abilities to properly function in their personal and social roles as a parent, partner or employee [5]. Stroke rehabilitation is a cyclic process which includes: assessing the needs of the patient, defining realistic and attainable goals, interventions or activities to achieve the goals and reassessment of the progress against the goals [6]. Rehabilitation is provided by an interdisciplinary team of health care professionals, including nurses, physical therapists, occupational therapists and other professionals, who support the patient to regain abilities that were lost. For the patient this is a time-intensive, effortful and often exasperating process [5, 7]. There is strong evidence that task-oriented training aiming to target functional tasks and ADL’s can assist the natural recovery pattern of functional recovery [6]. Task-specific and context-specific training are well accepted evidence based principles in stroke rehabilitation as well as the principle that increased intensity of training facilitates recovery [6, 8, 9]. Goals for training need to be relevant for the patient and occur in the patient’s environment, preferably his home surroundings. Generally, the literature emphasizes that patients with stroke need more rehabilitation training [8, 9].

Neuroscience nurses in stroke care are increasingly adapting to Evidence Based Practice integrating the best available evidence from well-designed studies with clinician’s expertise and with information about patient preferences and values in making the best clinical decisions [10]. Although many Interdisciplinary Stroke Practice Guidelines have been developed for the rehabilitation and management of patients with stroke, these guidelines are often not routinely incorporated into daily nursing practice. Among the reason for this is the fact that these guidelines often lack information about early detection of problems using valid and reliable instruments and interventions relevant and feasible for nurses to use in the daily context of stroke care and are not routinely incorporated into the daily patient care [4, 11, 12]. In an attempt to provide information on various important areas in stroke care, nurses, patients and health care professionals in Iceland and the Netherlands collaborated in developing the Clinical Nursing Rehabilitation Stroke Guideline Stroke (CNRS-Guideline) [13]. Systematic reviews were conducted on interventions and instruments feasible for nurses to use in following areas: mobility and ADL [9], communication and aphasia [3], depression (in patients with/without aphasia) [14, 15], falls [16], neglect [17], self-efficacy [18]. A feasibility study provided evidence for the usability of this guideline for patients and nurses in Dutch stroke settings [19]. Continuing work is taking place and studies are conducted with nurses on identification of symptoms of depression in patients with stroke [20, 21] and aphasia [22, 23], neglect and how to develop and use technical applications in the rehabilitation of patients with stroke residing at home. Based on this work, the Stroke Nursing Guideline (SNG) was developed and adapted including recommendations targeting among other important elements like mobility and ADL, falls, depression, pain and education of patients and family [24].

Nurses, as key members of the rehabilitation team, provide nursing specific rehabilitation through the continuum of care [8, 9]. They train patients in activities of daily living, as training needs to be functional, task oriented as well as context specific [6, 8, 9]. As patients with stroke need more training, they play an essential role in creating more opportunities for patients to exercise and practice functional tasks outside and in-between formal therapy sessions [9]. Accordingly nurses need to maximize their contribution in activation of patients and integration of functional and task oriented training exercises in simple activities, targeting mobility and ADL in the context of daily nursing care in order to increase the intensity and duration of rehabilitation exercise and training.

Painful shoulder is a common, complex and distressing complication after stroke which interferes with patients' recovery. Many patients experience painful shoulder in the early stage of stroke, which continues into the chronic stage, with an incidence ranging from 12 to 58% [25]. Although various therapeutic treatments have been developed, outcome studies show contrasting findings [25, 26].

Depression is a frequent complication after stroke affecting up to one third of patients [27]. Depression after stroke negatively impacts patients’ participation in rehabilitation, leads to worse functional outcome [28, 29] and higher mortality [30]. Although various guidelines recommend screening for depression in all stroke patients [4], depression after stroke remains unrecognized, undiagnosed and under treated [28]. Nurses routinely screen patients for depression which increases the early recognition of depression [31] and they effectively identify depression after stroke using the Patient Health Questionnaire [20, 21, 32].

Falls are common among stroke patients with prevalence ranging from10 to 73% [16, 33, 34]. The various risk factors for falls reported include: instability when walking, weakness of the lower leg muscles, urinary incontinence, frequent need to go to the toilet, confusion, depression and medication [16], a Barthel Index score below 15, time since stroke longer than 12 weeks, first fall associated with visuospatial neglect [35] older age, increased length of stay [36], greater stroke severity, history of anxiety, history of fear of falling [37], lower functional status and lower cognitive status [38]. Although moderate evidence was found for the ability of instruments to predict risk of fall in patients after stroke, the literature recommends preventive screening for risk of falls and to provide preventive measures for risk of falls in all phases after stroke [16, 33–38].

Education is an important aspect in the care of patients and families during the stroke recovery [39]. Due to the complexity of the impairments and the huge changes in life after the stroke incident, patients and caregivers have diverse educational needs which often are not met [40]. Patients and caregivers reported that they need education about the clinical aspects of stroke, stroke prevention, treatment and functional recovery and caregivers also need information concerning moving and lifting patients, exercises, psychological changes and nutritional issues after stroke, that is tailored to their situation [40]. Lack of knowledge about stroke can lead to misconceptions, anxiety, fear, poor health status and emotional problems [39, 40]. Therefore patients and caregivers need more and thorough education, tailored to their needs, after the stroke.

The Medical Research Council emphasizes the importance of evaluating feasibility and implementation of complex interventions like guidelines, in terms of acceptance by health care professionals, the nursing staff knowledge and skills and the facilities needed for implementation [41, 42]. Feasibility is referred to as the quality of being useful and practical and involves study of the applicability or practicality, which can be assessed by considering the acceptability of the guideline to clients and staff administering it, the costs and the ease of integrating it into clinical settings [43]. Implementation is defined as the introduction of an innovation in daily routines, demanding effective communication, and removing obstacles [12]. Unfortunately, the literature shows that implementation of CPGs is often not achieved and not following the evidence-based CPGs leads to suboptimal care for many patients [12]. Despite the evidence found for the usability of the earlier CNRS guideline, the fact that it was extensive and included many recommendations was found difficult for implementation [19].

Based on this background the aim of this study was to investigate the implementation and feasibility of the use of a Stroke Nursing Guideline (SNG) focusing on mobility ADL, depression, pain, falls, education and discharge planning, used by nurses and auxiliary nurses in the daily care of patients with stroke and stating the following research questions: a) What is the difference in nursing staff documentation of the screening and application of interventions for activities of daily living, mobility, depression, pain, falls, patient education and discharge planning of patients who receive rehabilitation nursing care before and after implementing the SNG? b) What are the nurses’ and auxiliary nurses’ view on the acceptability of using the SNG in supporting the provision of daily nursing care? c) What are the nurses’ and auxiliary nurses’ views on barriers and facilitators to implementing and embedding the SNG within routine daily nursing care?

## Methods

**Fig. 1:**
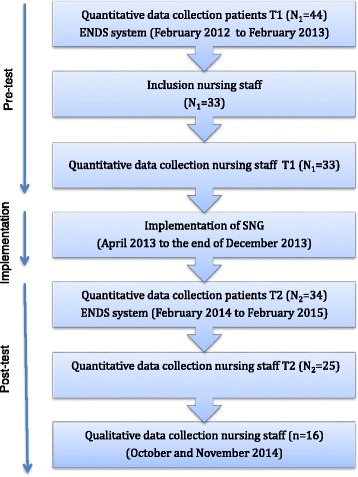
Flowchart of study design

This study used a sequential explorative mixed method design [44], including pre-test post-test measures [45] and focus group interviews [44]. The pre-test post-test was chosen to measure the difference in nursing staff documentation of the screening and application of interventions, whereas the focus group interviews explored the nurses’ and auxiliary nurses’ views of implementing and using the SNG. The study was conducted in three phases: In phase one (February 2012 to February 2013) pre-test retrospective patient record data were collected from: a) *patients’* electronic nursing documentation system (ENDS-system) on screening and application of key interventions in stroke care which included items focusing on: activities of daily living, falls, pain, depression, patient education and discharge planning, and b) *registered nurses* and *auxiliary nurses* answers on the Barriers and Facilitators Assessment Instrument (BFAI) [46] and the Quality Indicators Tool (QIT) reflecting the SNG. In phase two (April 2013 to the end of December 2013) the SNG was implemented using evidence based strategies including education and training, opinion leaders, posters and reminders [47, 48]. In phase three (February 2014 to February 2015), the posttest measurements were conducted with nurses and auxiliary nurses and patients assigned to the intervention group (February 2014 to February 2015). The focus group interviews were conducted with a subgroup of nurses and auxiliary nurses in October and November 2014 (Fig. 1). Hereafter, nurses and auxiliary nurses are generally referred to as nursing staff. To provide thorough reporting of the study both STROBE and COREQ statements were used (Additional file 1).

### Setting and participants

The study was conducted at neurology and rehabilitation wards of a university hospital in Iceland. Patient records were extracted from all patients diagnosed with stroke, older than 18 years of age, admitted to the acute neurological ward and subsequently transferred to the rehabilitation ward for 12 months prior to implementation and for12 months after implementation. Excluded were patients who died while admitted to the wards. Data were retrieved from 78 patients (34 in the pretest and 44 in the posttest).

All nursing staff, which included registered nurses and auxiliary nurses working on the participating wards (*N* = 40, nurses = 22 and auxiliary nurses = 18), were invited to take part in the study and signed informed consent. Thirty-three nursing staff responded to the pre-test questionnaires, whereas 25 responded to the post-test questionnaires (18 nurses/15 nursing auxiliaries/pretest and 13 nurses/12 nursing auxiliaries/posttest). Sixteen nurses and auxiliary nurses (*N* = 8 each group, respectively) took part in three focus group interviews.

### The stroke nursing guideline

The Stroke Nursing Guideline (SNG) aims to provide an overview of evidence based recommendations for the daily nursing care and rehabilitation of patients with stroke. The SNG was developed based on systematic reviews and studies focusing on following areas: mobility and ADL [8, 9], falls [16, 33–38, 49, 50], pain [25], depressive symptoms [14, 15, 20, 21, 28–32], education [39, 40, 51], as well as the CNRS-Guideline [13]. The authors, who all have extensive experience in stroke care and research, made the first selection of important interventions based on the literature, which were formulated as recommendations for the SNG.

Among important aspect of implementation and acceptability of new guidelines like the SNG is the fact that all professionals involved in the care of patients with stroke agree and support the guideline. Therefore, we approached a group of 20 interdisciplinary professional experts, to critically review the content, readability, layout and usability of the guideline. These experts included: nine nurses and of these seven worked on the wards, all with BSc degree in nursing and long experience in neuroscience nursing, of these four had a MSc degree and two had a PhD degree; six physical therapists, two occupational therapists; one psychologist; one rehabilitation physician and one neurologist. These professionals all agreed on the content of the guideline recommendations and their comments mainly focused on the readability, layout and usability of the SNG. There were no specific differences between the professionals in their views about the SNG and based on the expert feedback, the guideline was adapted and optimized.

The final SNG included a total of 23 recommendations focusing on assessment and therapeutic interventions categorized in the following areas: 1) activities of daily living and mobility and falls (14 recommendations), 2) pain/shoulder pain (3 recommendations); 3) depression (3 recommendations); 4) patient education (2 recommendations) and 5) discharge planning (1 recommendation). The guideline also included thorough instructions with photos on how to use the recommendations, with chapters on: background information, definition of concepts, flow-scheme of how to use the guideline, recommendations for the assessment of various outcomes including: mobility and activities of daily living using, the Functional Independence Measure (FIM) [52]; risk of falls using the Morse Fall Scale (MFS) [49]; shoulder pain using a visual analogue scale; depressive symptoms with Patient Health Questionnaire-9 (PHQ-9) [53, 54] and recommendations focusing on therapeutic interventions for the aforementioned areas as well as appendices with the instruments and instructions with photos on how to assist patients with mobility, exercises and positioning. The SNG guideline was made ready to use in a digital, online form as well as a 32 page manual including a plasticized card (pocket size) which was available for all staff.

### Data collection

Patient data were retrieved from the ENDS-system including: demographic and health care data: age, sex, living situation, height, weight, health history, the clinical diagnosis of stroke and the type of stroke (provided by a neurologist, based on a CT-scan or an MRI). Also, the following data concerning 37 items on screening and application of key interventions in stroke care were retrieved from the ENDS-system:

- *activities of daily living and mobility* (8 items) screened with the Functional Independence Measure (FIM) [52] within 72 h of admission, including diagnosis of mobility and ADL, evaluation of care, limitation in self-care, mobilization facilitation within 24 h, frequency of training exercises, walking exercises, training of ADL activities.
- *fall and fall risk* (1 item) screened within 72 h using the Morse Fall Scale (MFS) [49], consisting of six items reflecting risk factors of falling: (i) history of falling, (ii) secondary diagnosis, (iii) ambulatory aids, (iv) intravenous therapy, (v) type of gait and (vi) mental status. Total score ranges between 0 and 125 [49]. MFS had been translated into Icelandic (MFS-I) and piloted with the nurses to determine their understanding of wording of items. Interrater reliability was examined and the level of agreement was 84% (*K* = 0.68) [49].
- *pain assessment and pain treatment with special focus on shoulder pain* (14 items): Patients were asked about pain/shoulder pain and pain assessment was conducted using a visual analogue scale and the following interventions were provided: pain treatment (warm cold packages, massage), pain medication given, non-pharmacological treatment given, comforting, massage, relaxation, distraction, pain treatment never given, evaluation of pharmacological pain treatment).
- *patient screening for depressive symptoms* (4 items): Patients were asked about psychological distress, nursing diagnosis of depression, consultation of other professionals for the diagnosis and treatment. Depression was screened with the Patient Health Questionnaire-9 (PHQ-9). The scores are summed to produce a value ranging from 0 (no depression) to 27 (all symptoms occurring nearly every day [53, 54]. Symptoms of depression with the PHQ-9 was only screened in the posttest because no depression scale existed in the electronic documentation system prior to the implementation.
- *patient (and family) received education* (4 items) including standard information about stroke and rehabilitation, education brochure received, education repeated and tailored to the patient’s (and family) needs.
- *discharge planning* (6 items) which included: basic discharge planning using electronic patient record, quality discharge planning, patient discharge interview, social support recommended/planned, aftercare recommended/planned, written recommendations.

*Demographic data of the nurses and auxiliary nurses* were collected including: age, gender, education, experience/length of time working in stroke rehabilitation (0–2 years, 3–10 years, >10 years), current function (full time equivalent), courses on nursing stroke rehabilitation.

*Barriers and facilitators for implementation* were measured with the Barriers and Facilitators Assessment Instrument (BFAI) [46], with 27 questions, addressing four domains: *characteristics of the innovation* i.e. *the guideline; characteristics of the care provider, patient characteristics and context (organizational, social, political factors)*. The questions are positively as well as negatively formulated on a five-point Likert scale, ranging from 5 (strongly agree) to 1 (strongly disagree). The BFAI is a standardized and reliable instrument, with an item response of >90%, with each item having a distinctive character and was found to be useful for evaluating barriers and facilitators and with Cronbach’s alpha for the four domains ranging from 0.63 to 0.68 [46].

*The use of the guideline* was measured with a Qualitative Indicator Tool (QIT), developed by the authors, based on the SNG recommendations as and included 30 statements, for the nurses. The QIT statements focused on the main areas of the SNG: a) mobility and activities of daily living (7), b) falls (1), c) depression (9), d) pain/shoulder pain (5), e) patient education (5) and f) discharge planning (3) and inquired if the nurses provided care according to the SNG-recommendations and were phrased in line with the following statement as an example: “I conduct assessment of mobility and self-care activities on admission with a) the FIM-scale, b) the scale in the electronic patient health records, c) both FIM scale and the scale in the electronic patient health records”, which were scored on a five point Likert scale (almost never or <10% to very often or >90%). The face validity of the QIT was evaluated by a group of five experts and included clinical nurse specialists and nurse researchers with extensive experience in stroke nursing and rehabilitation, who reviewed the statements and concluded that the 30 statements were relevant for the daily care and rehabilitation of patients with stroke. Further psychometric testing of the QIT needs to be conducted.

#### Focus group interviews

Three Focus Group Interviews were conducted with eight nurses and eight auxiliary nurses after the implementation [44]. The interviews were chaired and conducted by a clinical nurse specialist in geriatric nursing, who is a seasoned researcher and has experience with focus group discussion, but was not involved in this study in other ways. An assistant observed and took notes on the interviews, how participants responded to questions and how the discussion evolved. The project manager (IB) invited participants to the interviews but did not take part in them. In the first interview seven nurses (*N* = 2) and nurse auxiliaries (*N* = 5) took part, in the second interview four nurses (*N* = 4) and no auxiliary nurses took part, whereas in the third interview five nurses (N = 2) and nurse auxiliaries (*N* = 3) took part. An interview guide was used to guide the interviews. The findings of the previous interviews were used to guide discussion in the subsequent interviews (Additional file 2).

### Procedure

#### Phase 1. Pre-test

Quantitative data of the pre-test group of patients were collected from the Ends-system prior to the implementation of the SNG. Pre-test measures of the nurses and auxiliary nurses were collected as well, after presenting the study including the purpose and procedures in a meeting with the nurses, nurse auxiliaries and managers of the ward.

#### Phase 2. Implementation

The SNG was implemented in the course of nine months using the following implementation strategies which were based on the literature [47, 48]: a) *Stroke Nursing Guideline:* all the registered nurses and auxiliary nurses received both a printed and plasticised version as well as a digital version. b) *Education and Training sessions:* All the registered nurses and auxiliary nurses as well as other professionals were invited to take part in one of two, four hour education and training session in how to use the recommendations, the screenings instruments and interventions recommended. This training was strongly recommended for the nurses and the nurse auxiliaries. c) *Opinion leaders*: seven nurses (5 registered nurses and 2 auxiliary nurses) took on the role of an opinion leader. The opinion leaders were experts in the content and application of the guideline. They followed up on the implementation of the guideline, observed if recommendations were used and gave advice to other colleagues concerning the application of the recommendations. d) *Posters and reminders:* Posters and reminders were placed on the walls of the wards to remind the nurses on using the guideline and e) E-mails: Regular e-mails were sent to all the registered nurses and auxiliary nurses explaining the intervention protocol and the recommendations.

#### Phase 3. Post-test

After the implementation period, the post-test data collection took place. The same data were collected as in the pre-test. In addition, focus group interviews were conducted with a subgroup of nurses and auxiliary nurses. The focus group interviews took place in a quiet room within the nursing science department and not within the hospital wards.

### Data analysis

Quantitative data were analyzed using descriptive statistics to describe the characteristics of the patients including means (SD), medians (IQR) or n (%). Frequencies and percentages were reported for the recommendations used, perceived barrier quality indicators were analyzed and reported for both control and comparison group. Associations were calculated for specific patients’ health problems and specific recommendations using Fisher’s exact Test (2-sided) and Spearman’s rho. All data were assessed for normality, which was taken into account when choosing the appropriated statistical method used. For analyzing the Perceived barriers and facilitators measured with the BFAI, the items 4–15 and 17–27 were revised so that a higher score reflected positive and low score negative view of participants. A *p*-value of <0.05 was considered significant. The SPSS version 20 (SPSS inc., Chicago IL, USA) was used.

Qualitative data analysis was carried out with content analysis (44). The first stage in the qualitative analysis process involved transcription of the interviews. The transcripts were studied repeatedly by two researchers (IB/HJ). Following the transcription, the content was checked for accuracy, after which the data were analyzed. The first level of analysis involved grouping that under broad headings in the interview guide and data were categorized to answer the research question(s) by extracting the quotes from the transcribed interviews. The authors read and reread the transcribed interviews, initial themes were identified using open coding of the data. Differences in themes were resolved by discussions (IB/HJ/TBH). Member checking was employed to ensure content validity by obtaining agreement from participating nurses on a summary of the focus group findings.

Quantitative and qualitative results were integrated after data analysis [44], results of these data were presented separately but integrated in the discussion section.

### Research ethics

The study was conducted in accordance with the Declaration of Helsinki (revised form, Seoul 2013) [55]. The Hospital Ethics Committee (1909201223–2012, 0411201323–2012, 1,701,201,423–2012,1,603,201,523–2012, 1,007,201,523–2012, 23/2012), the Ethics Committee of the CEO of Medicine (3,005,201,516, 16LSH-14,1,203,201,516), Human Resource Council of the hospital (2505201216) and the Data protection Authorities (2,012,050,710, 2,014,010,073, S6717–2014) approved the study. All the nurses and nursing auxiliaries consented to participation and the use of direct quotes in this paper by signing an informed consent form.

## Results

### Patients and nurses characteristics

In total data were extracted from 78 patients. Analysis was based on data from 44 patients in the pre-test group (T1) and 34 patients in the post-test group (T2) and Patients in both groups were comparable on main demographic variables, except that the patients in the post-test group were younger (*p* = 0.051) (Table 1). A total of 33 nursing staff were included in the study and of these 18 were registered nurses (54%) and 15 were nursing auxiliaries (46%). Of the group 25 (76%) worked on the rehabilitation ward whereas eight (24%) worked on the neurological ward. Most of the staff worked part-time (Table 2).

**Table 1:** Characteristics of patients

	Pre-test (*N* = 44)	Post-test (*N* = 34)	*p*-value
	Group	Group	
Gender (n,%)			0.246
Men	29 (66)	18 (53)	
Women	15 (34)	16 (47)	
Age (M, SD)	65.5 (13.12)	58.2 (17.90)	0.051
Disease diagnosis (n,%)			
Hemorrhage	11 (25)	8 (24)	0.881
Infarct	33 (75)	26 (76)	
Living situation (n,%)			0.763
Single/lives alone	11 (25)	10 (30)	
Married/cohabiting	32 (75)	23 (70)	
Employment status prior to admission^a^(n,%)			0.438
Full employment	12 (29)	10 (35)	
Part time	2 (5)	1 (3)	
Not working	2 (5)	1 (3)	
Retired	20 (49)	9 (31)	
Disability benefits	5 (12)	8 (28)	
Nationality (n,%)			
Icelandic	44 (100)	31 (91)	0.044
Non-Icelandic	0 (0)	3 (9)	
Length of hospital stay days (M, SD)			
Neurological ward	17.8 (13.10)^b^	14.7 (7.162)	0.225
Rehabilitation ward	58.0 (48.27)	58.8 (56.71)	0.135

^a^Missing data, ^b^2 patients excluded due to unusual long acute phase

**Table 2:** Characteristics of nurses and auxiliary nurses (*N* = 33)^a^

	N (%)
Ward (n, %)	
Rehabilitation	25 (76)
Neurological	8 (24)
Profession (n, %)	
Registered nurses	18 (54)
Auxiliary nurses	15 (46)
Age (years) (n, %)	
< 34	10 (30)
35–44	3 (10)
45–54	5 (15)
55–64	10 (30)
> 65	5 (15)
Highest educational degree/diploma (n, %)	
Nursing Bachelor of Science/Diploma	14 (43)
Postgraduate nursing program	5 (15)
Nursing auxiliary program	12 (36)
Postgraduate nursing auxiliary program	2 (6)
Full time equivalent work (FTE) (n, %)	
100%	5 (16)
50–90%	24 (77)
40–49%	2 (7)
Working experience in nursing (years) (n, %)	
< 4 years	1 (3)
1–5	7 (21)
≥6	25 (76)
Working experience in stroke rehabilitation (years) (n, %)	
0–2	6 (19)
3–10	13 (42)
>10	12 (3)
Nursing stroke rehabilitation courses attended (n, %)	
Mobility/self-care	19 (58)
Psychological care	13 (39)
Patient education	12 (36)
Falls	12 (36)
Pain	15 (45)
Other	2 (6)

^a^There is lack of responses on all items, varying between 2 and 4

### Difference in documentation of SNG key interventions before and after implementation

Documentation of the 37 items on screening and application of key interventions in stroke care, was improved in 23 items after implementation. Significant improvement was found on the six following items: a) three items in ADL and mobility: Assess with FIM < 72 h of admission (*p* = 0.002), Mobilization facilitation within 24 h (*p* = 0.024), Training of ADL (*p* = 0.022) and b) three items on patient education: Patient education (*p* = 0.001), Educational brochure provided (*p* = 0.000) and Education repeated (*p* = 0.049). No change was found in the documentation of five items (4 pain variables, 1 depression). Significant worse documentation was found for the item Patients asked about pain (*p* = 0.012), whereas the worse documentation on the remaining eight items was non-significant (3 ADL, 4 pain, 1 depression) (Table 3).

**Table 3:** Comparison of documentation of Quality Indicator Tool items of the Stroke Nursing Guideline

	Pre-test Group (N = 44)		Post-test Group (N = 34)		*p*-value^a^
	No (%)^b^	Yes (%)	No (%)	Yes (%)	
Mobility and Activities of daily living (n, %)					
Assess. with FIM < 72 h of admission	33 (75)	11 (25)	14 (41)	20 (59)	*0.002*
Nursing diagnosis of mobility	4 (9)	39 (91)	1 (3)	33 (97)	0.261
Evaluation of care	33 (75)	11 (25)	28 (85)	5 (15)	0.292
Limitation in self-care	17 (39)	27 (61)	9 (26)	25 (74)	0.258
Mobilization facilitation <24 h	19 (47)	21 (53)	7 (22)	25 (78)	*0.024*
Frequency of training exercises	12 (35)	22 (65)	10 (39)	16 (61)	0.180
Walking exercises	4 (14)	25 (86)	4 (17)	20(83)	0.778
Training of ADL activities	12 (30)	28 (70)	2 (7)	26 (93)	*0.022*
Falls (n, %)					
MORSE screening	34 (77)	10 (23)	21 (62)	13 (38)	0.306
Pain and pain treatment (n, %)					
Patients asked about pain	10 (23)	34 (77)	17 (50)	17 (50)	*0.012*
Pain diagnosis	10 (23)	34 (77)	6 (18)	28 (82)	0.582
Pain assessment with a scale	23 (74)	8 (26)	16 (73)	6 (27)	0.905
Fixed pain treatment	7 (21)	26 (79)	9 (39)	14 (61)	0.144
PN pain treatment	10 (30)	23 (70)	8 (32)	17 (68)	0.890
Non-pharmacological pain treatment	22 (73)	8 (27)	12 (55)	10 (45)	0.159
Comforting	42 (96)	2 (4)	32 (94)	2 (6)	0.589
Massage	43 (98)	1 (2)	31 (91)	3 (9)	0.217
Electrotherapy	44 (100)	0 (0)	34 (100)	0 (0)	–
Ankle splint	43 (98)	1 (2)	34 (100)	0 (0)	0.564
Relaxation	44 (100)	0 (0)	34 (100)	0 (0)	–
Distraction	44 (100)	0 (0)	34 (100)	0 (0)	–
Pain treatment never given	28 (78)	8 (22)	21 (78)	6 (22)	1.000
Evaluation of pain treatment	4 (14)	25 (86)	7 (33)	14 (67)	0.097
Depressive symptoms (n, %)					
Psychological distress diagnosis	18 (41)	26 (59)	15 (45)	18 (55)	0.690
Assessment with PHQ9	–	–	29 (88)	4 (12)	–
Identification of depressive symptoms	–	3 (7)	–	3 (9)	–
Consultation other professionals for the diagnosis and treatment	25 (58)	18 (42)	13 (38)	21 (62)	0.083
Patient teaching (n, %)					
Patient education	37 (84)	7 (16)	15 (47)	17 (53)	*0.001*
Educational brochure	40 (95)	2 (5)	15 (48)	16 (52)	*0.000*
Education repeated	30 (91)	3 (9)	30 (91)	14 (19)	*0.049*
Participation in teaching sessions	39 (89)	5 (11)	27 (82)	6 (18)	0.397
Discharge planning (n, %)					
Electronic Patient Record	11 (32)	23 (68)	18 (42)	25 (58)	0.393
Quality Discharge Planning*	7 (30)	16 (70)	22 (85)	4 (15)	*0.001*
Discharge Interview	22 (69)	10 (31)	43 (100)	0 (0)	*0.000*
Social support	7 (22)	25 (78)	14 (38)	23 (62)	0.151
Advice follow-up	20 (63)	12 (37)	36 (92)	3 (8)	*0.002*
Written infomation & recommendation	25 (81)	6 (19)	42 (100)	0 (0)	*0.004*

a) p-value calculated with Chi square test; p-value cursive indicates significant difference between groups;

b) No = very limited information documented; Yes = somewhat good and very good, with relevant information

### Difference in the use of the SNG measured with the quality indicator tool

The nurses’ use of the guideline measured with the 30 item QIT, showed enhanced use on 20 indicators, six of which the improvement was significant (Table 4). Improvement in use of the guideline was shown in seven indicators (of eight) on *Mobility and ADL*, with significant improvement in one item, namely *Assist and supervise patient with exercises according to physical therapists recommendations (p = 0.023)*. Improvement was shown in four (of eight) indicators on *Depression*, with significant improvement for three items: *Assess symptoms of depression with a depression scale (p = 0.033), Take time to talk with patient (p = 0.046), Take time to talk with family (p = 0.046)*. Non-significant improvement trend was shown in four (of five) indicators on *pain* as well as on two (of five) indicators on *Patient education* indicators. Improvement was shown on two (of four) indicators on *Discharge planning* and of these significant improvement was found for the indicator *Document discharge planning in patient electronic health records*. On the remaining 10 indicators no improvement was found (Table 4).

**Table 4:** Difference in nurses’ application of 30 quality indicators before and after implementation of the Stroke Nursing Guideline (*N* = 14)

	Pre-test group M (SD)	Post-test group M (SD)	*p*-value
Mobility and activities of daily living			
Assess mobility and self-care capabilities on admission to the ward with			
a) FIM scale	1.818 (0.982)	1.727 (0.273)	0.655
b) scale in patient electronic health records	2.909 (1.640)	3.091^a^ (1.446)	0.672
c) both FIM scale and scale in electronic patient health records	1.750 (1.036)	2.000^a^ (1.195)	0.157
Assist patient with getting in and out of the bed on the first shift on the ward	4.077 (0.760)	4.231^a^ (0.726)	0.157
Assist and supervise patient to transfer between bed and chair	4.462 (0.877)	4.615^a^ (0.650)	0.157
Assist and supervise patient with exercises according to physical therapists’ recommendations	3.692 ((1.032)	4.308^b^ (0.947)	*0.023* ^*b*^
Assist patient in ADL and coach transferral of exercises into ADL	4.308 (1.109)	4.385^a^ (0.768)	0.739
Assist patient with hemiplegia to exercise the paralysed arm	3.462 (1.050)	3.615^a^ (1.193)	0.564
Assist patient with hemiplegia to make personal goals in writing if needed	3.846 (1.068)	3.769 (1.166)	0.705
Falls			
Assess risk of falls with MORSE scale	2.846 (1.519)	3.231^a^ (1.092)	0.129
Pain			
Prevent shoulder pain by comforting the paralysed arm	4.846 (0.376)	4.923^a^ (0.277)	0.317
Teach patient how to prevent shoulder pain	4.000 (1.000)	4.308^a^ (1.109)	0.234
Teach family how to prevent shoulder pain	3.417 (0.669)	3.667^a^ (0.888)	0.317
Grade patient’s pain by pain scale	3.692 (1.109)	3.385 (0.961)	0.157
Use non-pharmacological pain interventions	3.250 (1.056)	3.833^a^ (0.835)	0.107
Depression			
Assess symptoms of depression with a depression scale	1.231 (0.599)	1.846^b^ (0.801)	*0.033* ^*b*^
Refer patient to a psychologist due to depression	2.857 (1.351)	3.071^b^ (1.207)	0.438
Refer patient to other HCPs e.g., chaplain or social worker	2.750 (1.139)	2.500 (0.798)	0.180
Provide emotional support e.g., with active listening	4.429 (0.646)	4.214 (0.699)	0.083
Encourage patient to believe in own ability by identifying his/her strength and progress in the rehabilitation	4.643 (0.497)	4.286 (0.611)	0.025* ^*b*^
Coach patient to relax e.g., by listening to music	3.167 (1.267)	3.500^a^ (1.382)	0.305
Take time to talk with patient	4.143 (0.663)	4.429^b^ (0.514)	*0.046* ^*b*^
Take time to talk with family	4.071 (0.730)	4.357^b^ (0.633)	*0.046* ^*b*^
Patient teaching			
Give patient individualized teaching material upon admission	2.583 (1.240)	2.833^a^ (1.193)	0.048
Secure patient teaching about stroke, its consequences and planned diagnostic tests and treatment	3.071 (1.269)	3.429^a^ (1.089)	0.227
Secure family teaching about stroke, its consequences and planned diagnostic tests and treatment	3.077 (1.188)	3.615^a^ (0.650)	0.052
Teach patient about the importance that the family participates with patient in rehabilitation	3.692 (1.437)	3.846^a^ (1.068)	0.564
Teach family about the importance of their participation with patient in rehabilitation	3.667 (1.371)	3.917* (1.165)	0.257
Discharge planning			
Document discharge planning in patient electronic health records	2.833 (1.267)	3.917^b^ (1.084)	*0.012* ^*b*^
Assess patient’s need for social support after discharge	4.214 (0.893)	4.143 (0.864)	0.739
Assess mobility and self-care capabilities in discharge planning with			
a) FIM scale	1.727 (1.272)	2.000^a^ (1.095)	0.317
b) scale in patient electronic health records	2.500 (1.650)	2.700^a^ (1.494)	0.480
c) both FIM scale and scale in electronic patient health records	2.125 (1.356)	1.750 (1.165)	0.180
Conduct discharge planning interview, provide personalized information	3.000 (1.291)	2.923 (1.256)	0.739

^a^=differences, ^b^ = significant differences

In the analysis of the focus group interviews the following six themes emerged: *Improved quality of care, Content known to staff, Convenient and concise, More use of instruments, More consistency, Illustrative and instructive.* The focus group interviews showed that the nurses and auxiliary nurses viewed the use of the guideline to improve nursing care. They knew the content of the guideline, used it and found the guideline practical and easy to use. The use of the SNG made them focus more on specific issues like depression and falls and provided accurate and systematic way to evaluate and communicate about patients’ progress. It provided consistency in care as they provided care and exercises in the same way, with consistency in intensity, frequency, with more rigorousness and better use of ergonomics than before. They found the guideline layout, including photos and diagrams, to be illustrative and instructive for patients, who are mobilized and cared for in a convenient and consistent way. Family members were more trustful in that the patients received optimal care. At the end of the focus group interview, the nurses and auxiliary nurses participating were individually asked to rate their view of the general usefulness of the SNG on visual analogue scale (ranging from 1 indicating not useful to 10 indicating very useful) which was valued with a mean score of 7.7 (range 5.5–9.0)(Table 5).

**Table 5:** Nurses view of the usefulness of the Stroke Nursing Guideline and Implementation process (N=16)

Mean		Themes	Descriptions	Quotes
Usefulness of the Stroke Nursing Guideline				
Mean = 7.7 Range = 5.5–9.0	1.	*Improved quality of care*	This theme described how the SNG generally improved nursing care generally.	“The SNG has improved the way we work, especially when assisting patients with moving and positioning”. “The SNG has both improved the care, we think more about how we approach patients and how we help them with movement and ADL”. “We do not only think about physical care but also psychological care, like depression”. “We ask patients more about how they feel, − their psychological well being”. “We make much more use of scales now”. “We think more about the emotional par now and not only about the phhysical”.
	2.	*Content known to staff*	The content of the SNG was generally known to staff and already used to an extent in daily care.	“The SNG had not so much new things in it, but very good to have everything set up like this”. “Some things were known to us already, but others are new, − like more emphasis on scales and of course depression”.
	3.	*Convenient and concise*	The SNG was convenient and teh text was concise, effortless to read, handy and practical, particularly for new staff and students.	“The recommendations are convenient and really very practical and fit very well with how we work on the wards”. “The guideline is very easy to use. They (the recommendations) are not so extensive, they are short and easy to use”. “The guideline is very easy to use”. “We have had much new nursing staff and then it is very good to have the guideline”.
	4.	*More use of instruments*	Screening tools make staff focus more systematically on respective components e.g., depression, anxiety, risk of fall, and nutritional status, to be accurate in communicating about patients‘symptoms, as well as to evaluate patients‘progress.	“We use instruments more, especially the PHQ-9”. “We are using the scales much more now with the guideline”. “Now we use scales for most things like walking ability, falls, depression”. “The scales are very easy and practical to use”.
	5.	More consistency	The SNG makes staff do things the same way, which is a quality issue, and with consistent intensity and frequency e.g., in doing physcial exercises with more rigorousness in the evenings and weekends.	“After following SNG and the training, we are all working in the same way, − there is much more consistency in how we move patients”. “It is good that we are all working in the same way. For example when we are taking patients out of bed. Before the guideline we did this very differently”.
	6.	*Illustrative and instructive*	Concenring the layout of the SNG, the photos and diagrams are illustrative and instructive a) for staff who uses better ergonomics and b) for patients who are mobilized in a convenient and consistent way and c) for family members who can trust that patients receive the right care.	“We use the photos to show patients and family when patients go home for the weekend”. “Good positions for in bed or when sitting, but also concerning the pain”. “We can use the SNG much more with family”.
Implementation process				
Mean = 7.5 Range 6.0–8.5	1.	*Nursing rehabilitation defined and integrated*	Through the SNG, essential components of nursing rehabilitation have been defined and integrated into daily nursing care, e.g., going to the toilet is an opportunity to exercise stand up and sit down, rather than only being the fullfilment of a basic human need.	“The SNG is very compact. There is not so much new, − but it is much more clear now. Very clear guideline”. “All these elements of nursing, like moving and ADL, screening for falls, mobility or depression, which were somehow hidden, are more clear now”. “Integrating exercise into daily activities is so good for the patients”. “We now say: Do you need to go to the toilet? Yes, great! That is exercise (laughs)”. “We now do much more of general training, − activating patients”.
	2.	*Physical exercise Individualized*	Physcial exercise guidelines have made individualized instructions from physical therapists less needed.	“The mobililty ADL part of the guideline is very good, gives good instruction on how to mobilize patients. Also positioning, − especially the arm”. “Very good to have the photo‘s on mobility and positioning, − we are becoming much better in helping and instructing on how to move and do excercies”.
	3.	*Enhanced patient and family teaching*	Enhanced patient and family teaching, with particularly good teaching material (booklet), bringing forth a request for structured family interviews.	“It is much better to teach patients and family about mobility and integrating exercises into daily activities when having this written down and digital”. “I like to have this in a printed map, which you can take with you and show to patients and family”.
	4.	*Coherent and consistent leadership*	Leadership of the charing group during implementation of the SNG was coherent and consistent.	“The implementation went very well”. “The implementation was well led by the chairing group, − they did a very good follow up on things”. “They (the charing group) really were in charge of things”.
	5.	*Improved staff education*	The SNG resulted in good/improved staff education, which needs to be repeated consistently throughout the care continuoum.	“The educational and training sessions for staff were very good, − but it needs to be repeated regulary”. “It is much better to have an active training like this, − you need to do the things and not only read about them”. “We need to have the training sessions repeated regularly to refresh things, − you tend to forget”.
	6.	*Less visible nursing care received attention*	Through the SNG, previous less visible aspects of nursing care have received attention and recognition among all staff, particularly its contribution to the success of patient rehabilitation.	“Posters with photo‘s on positioning and mobilizing of patients have been put on the walls for patients and famly as well for staff. Nursing and what we do in rehabilitation is now more visible for all staff”. “The guideline has made elements of nursing care much more visible to other staff as wel”. “What we nurses are doing in rehabilitation, like mobilizing and stimulating patients to exercise is now much more visible to the other staff”.

### Nursing staff view of the implementation process

Facilitating characteristics for change were significantly less for two of the four subscales, namely Innovation (*p* = 0.019) and Context (*p* = 0.001) on the BFAI, whereas no change was found for Professional and Patient subscales (Table 6). Contrary to these results, the nurses and auxiliary nurses reported positive experiences, when asked to rate the success of implementation on visual analogue scale (ranging from 1 indicating not successful to 10 indicating very successful) which was valued as successful with a mean score of 7.5 (range 6.0–8.5). They maintained that the implementation brought a totally different view on mobilization in daily care (Table 5). In the analysis of the focus group data, the following six themes emerged: *Nursing rehabilitation defined and integrated, Physical exercise Individualized, Enhanced patient and family teaching, Coherent and consistent leadership, Improved staff education* and *Less visible nursing care received attention.* The focus group interviews showed that the nurses and auxiliary nurses found that throughout the implementation consistent and coherent leadership was provided. They found that essential components of rehabilitation had been defined and integrated into daily nursing care (standing up and sitting down, going to the toilet). The exercise guidelines made individual instructions from other professionals less needed. There was enhanced patient and family teaching, good teaching material, and consistent and good staff education. Previous less visible aspects of nursing care, after implementation, received attention and recognition among all staff. Of particular significance was the contribution this makes to the entire rehabilitation of patients with stroke (Table 5).

**Table 6:** Difference on the Barriers and Facilitators Assessment Instrument before and after. implementing the Stroke Nursing Guideline (*N* = 20)

	Item	Pre-test group	Post-test group	*p*-value
	(*N* = 27)	M (SD)	M (SD)	
Innovation	6	4.017 (0.492)	3.755 (0.509)	*0.019* ^*a*^
Professional	10	3.874 (0.445)	3.821 (0.675)	0.074
Patient	6	3.392 (0.630)	3.415 (0.563)	0.055
Context	5	2.632 (0.547)	2.474 (0.542)	*0.001* ^*a*^

^a^A *p*-value cursive indicates significant difference between groups

## Discussion

This study investigated the implementation and feasibility of a newly developed Stroke Nursing Guideline using electronic data on patient outcomes before and after implementation and data from nursing staff on barriers and facilitators for implementation, quality indicators before and after implementation of the SNG and the views and opinions of nursing staff towards the guideline. In this way we aimed to gain better understanding of the implementation, use and feasibility of the SNG in daily care of hospitalized patients with stroke. Both the documentation and quality indicators showed that the nursing staff applied more mobility and ADL interventions, which included screening functional status and providing patients with exercise and training, and interventions focusing on education of patients and family all of which was supported by the qualitative findings. Also, satisfactory attention was paid to observing and assessing patients for the symptoms of depression which was also supported by the qualitative findings.

The feasibility and usefulness of the SNG, both the quantitative and qualitative findings showed that the nursing staff found the SNG useful. The findings of the focus group interviews also showed that the SNG recommendations were practical and easy to use and that it improved nursing care. The guideline layout was also illustrative and instructive for patients and family members.

Contrary to what was anticipated the facilitating factors on the BFAI instrument after implementation were lower for the subscales of “Innovation” and “Context” and no change was found for the “Professional” and “Patient” subscales. The qualitative findings, however, showed relatively positive experiences. The nursing staff judged the implementation to be successful, which was rated with the mean score of 7.5. They reported that they had taken an active part in the implementation. The implementation had brought a totally new view on mobilization in daily care and they found that consistent and coherent leadership had been provided during implementation. Through the SNG, essential components of rehabilitation had been defined and integrated into daily nursing care. Less visible aspects of nursing now received more attention and recognition. Explanation for this mismatch may be found in the questions of the BAFI which generally refer to the context and professional issues on the ward. At the time of the implementation of the guideline, severe organizational and budgetary restrictions were taking place.

The study showed improved documentation by the nursing staff after implementation of the SNG in 23 items focusing on screening and application of interventions. Significant improvements were found in three items focusing on *Mobility and ADL*. Likewise, parallel findings were found in that the nurses used the SNG more on the items *Mobility and ADL* indicators and with significant improvement in *Assisting and supervising patients with exercises according to physical therapists recommendations,* which was in line with the scores on the QIT. This was as well supported by the qualitative findings of the focus group interviews. The SNG provided consistency in care, particularly as the patients did exercises in the same way and there was more consistency in intensity and frequency of exercises. This indicates that the nursing staff generally paid more attention to mobility and ADL, conducting mobility assessments and actually mobilizing patients and providing them with exercises. This finding is in line with the findings of our earlier study investigating the feasibility of the CNRS-Guideline implemented in various stroke settings in the Netherlands [19]. It is however important to note that our study measured the documentation by the nursing staff and not the patient outcomes. However, various studies have shown that health care professionals pay limited attention to mobilizing patients with stroke. A recent intervention study comparing the amount of time spent in moderate-to high physical activity of stroke survivors on rehabilitation ward and acute stroke wards in Sweden showed that the amount of time spent in moderate-to high physical activity ranged between 24% on a rehabilitation ward and 23% on acute ward with no difference between the two groups. Compared to those in the acute setting, participants in the rehabilitation setting spent less time lying in bed, more time sitting supported out of bed, less time in their bedroom, and more time with a therapist (all adjusted *P* < .001) [56]. An observational behavioral mapping study, showed that stroke patients different medical wards were found inactive and alone for 19 to 15% of the time during the day and spent 46% of the time in therapeutic activities and 31% of the time in non-therapeutic activities. The family was present with patients 50% of the time during the day. The family presence with the patient and the patient’s moderate dependence in daily activities were positively associated with their activity levels. The authors concluded that the presence of family members with the patients during hospital stay may be a significant resource for encouraging patients to be more active [57]. Two smaller studies showed that patients in Dutch nursing homes were inactive and alone for up to 49% and 60% of the day [58, 59]. Therapeutic time use was significantly related to improved functional status; patients with higher functional status spent more time on therapeutic activities [58]. It is highly important that nursing staff activate patients and provide them with opportunities to do exercises in between physical therapy and occupational therapy training sessions and the findings of this study suggest that the SNG is exactly facilitative of that.

Depressive symptoms were only measured post-intervention as the nurses did not conduct screening of depression prior to the implementation of the SNG. After implementation of the SNG, the application of the SNG recommendations was quite satisfactory as three out of four items on the QIT were used. This was supported by the qualitative findings of the focus group interviews which showed that the nurses paid more attention to depression and they used the PHQ-9 for screening. In our earlier study, investigating the feasibility of the CNRS-Guideline implemented in various Dutch stroke settings, we found that the nurses acknowledge the importance of assessing and acknowledging the symptoms of depression, but they rarely used recommended instruments for screening depression or evidence based interventions [19]. Depression after stroke is frequent and strongly impacts patients’ recovery as patients have worse functional outcome, lower quality of life and are at more risk of dying [27, 28, 60, 61]. There is however growing evidence for the beneficial effects of physical activity [62], self-efficacy [18, 63] and social support [64] all of which can be used by nurses in the daily care of patients with stroke.

This study shows that after the implementation of the SNG the nurses reported enhanced patient and family teaching and that they provided good teaching material that focused on patients and family. This extends previous research which has pointed to the importance of patient and family education albeit with a lack thereof [40]. A meta-analysis including 21 trials (2289 patients and 1290 caregivers) and assessing the effectiveness of education provided to patients with stroke and their caregivers, provided evidence that education improves patient and caregiver knowledge of stroke, aspects of patient satisfaction, and reduces patient depression scores. The authors recommend that, although the best way to provide education is still unclear, there is some evidence that strategies that actively involve patients and caregivers in education and includes planned follow-up for clarification and reinforcement have a greater effect on patient mood [65].

Although the study showed improved documentation and use of the SNG on items focusing on mobility and activities of daily living, depressive symptoms, patient teaching and discharge planning, the results of the study show that the implementation and use of the SNG still can be improved on items focusing on pain or falls. The question remains as to why the other elements of the SNG were not as well applied. The nursing staff generally judged the guideline to be practical and easy to use. Earlier studies, however, have reported similar results. Metzelthin and colleagues [66] investigated the implementation of a nurse-led interdisciplinary primary care approach using a process evaluation and concluded that some parts of the program were insufficiently executed [66]. Similar findings were reported in a mixed method study investigating care delivery of a nurse-led intervention, where some time-consuming interventions were less often applied than other interventions [67]. A feasibility study of a fall-prevention program, where interventions that required more knowledge, communication and extra activities were implemented the least. The absence of materials and knowledge about falls prevention were important determinants of the non-implementation of certain interventions [68]. However, given the complexity of guidelines like the SNG, implementation is challenging and needs continuous education of nursing staff and other professionals. It is highly important to continuously monitor and evaluate the implementation and use of the SNG and to verify the extent to which the SNG recommendations are delivered as intended. Further research is warranted into the development and testing as well as implementation and translation of complex interventions like the SNG into the daily care of patients with stroke.

### Strengths and limitations

To appreciate the findings of this study, some limitations need to be considered. The fact that the study took place on only two wards within the same hospital and the fact that the sample of nurses participating was a small convenience sample, which was due to intense workload of nurses, unprecedented staffing shortages, including organizational changes occurring at the same time, and is in line with earlier studies [19, 69, 70]. Therefore, caution is indicated in generalizing the results of this study to other nurses in different organizational settings. However, the demographic data from both nurses and patients participating in our study do reflect the Icelandic population. Although we conducted thorough translation procedure of the instruments used no psychometric testing was conducted. The researchers participated in the development of the guideline and the implementation process, which could have limited objectivity. However, because of the quality assurances taken, the quality of the data can be ensured. The implementation strategies used was based on the literature, with active and multifaceted aspects, which benefited the study [44, 45]. The mixed method design provided rich data. The findings of the qualitative part were illustrative of the findings of the quantitative findings of the study to which they provided more depth.

Some may, however, consider the design of the study to be limited by the fact that we measured difference in the nurses documentation of SNG key interventions before and after implementation and not difference in patient outcomes. It is important to note that this study was not an outcome study, but a feasibility study investigating the usability of the SNG and documentation of interventions is an important parameter in measuring usability. Further, robust outcome studies are warranted to investigate the effects of the SNG on various patient outcomes including larger samples with a longer follow-up period.

## Conclusions

The findings of this study indicate that implementation of the SNG improved patient care as illustrated in the patient electronic documentation system, nurses answers on the Quality Indicators Tool and focus group interview with nursing staff. Most improvements were found on assessing mobility and ADL and patients were activated more and they as well participated more in exercise and training. The nursing staff gave more education to patients and families and they paid more attention to the symptoms of depression and screened patients for depression. Using the SNG, the essential components of rehabilitation were defined and integrated into daily nursing care. The nursing staff found the SNG feasible and that it was practical and easy to use and it improved nursing care. The guideline layout was illustrative and instructive for patients and family members. The nursing staff judged the implementation of the SNG to be successful and they generally took an active part in the implementation. The SNG needs to be further developed and robust research needs to be conducted to investigate the effects of the SNG on the outcomes of patients with stroke in various settings where patients with stroke reside. Thereby we may be able to improve the clinical outcomes of patients with stroke.

## Additional files

Additional file 1: STROBE and COREQ statements. (DOC 131 kb)

Additional file 2: Interview guide. (DOCX 16 kb)

## Abbreviations

ADL: Activities of Daily Living
BFAI: Barriers and Facilitators Assessment Instrument
CNRSG: Clinical Nursing Rehabilitation Stroke-guideline
CPG: Clinical Practice Guidelines
FIM: Functional Independence Measure
HJ: Helga Jónsdóttir
IB: Ingibjörg Bjartmarz
MFS-I: Morse Fall Scale-Icelandic
MSF: Morse Fall Scale
PHQ-9: Patient Health Questionnaire-9
QIT: Quality Indicator Tool;
SNG: Stroke Nursing Guideline
TBH: Thóra B. Hafsteinsdóttir
