# Supplementary material for: Implementation and feasibility of the stroke nursing guideline in the care of patients with stroke: a mixed methods study
Source: BMC Nurs. 2017 Dec 1;16:72. doi: 10.1186/s12912-017-0262-y (PMC5709925; doi:10.1186/s12912-017-0262-y)
Supplement: Supplementary file 1 — STROBE and COREQ statements. (DOC 131 kb) [file 12912_2017_262_MOESM1_ESM.doc]

Implementation and feasibility of the Stroke Nursing Guideline in the care of patients with stroke: a mixed methods study

Both STROBE and COREQ statements were used.

(both are here below in this one document).

This concerns mixed methods study. We use the Strobe statement for the reporting of the quantitative part of the study (not all items are relevant) and the COREQ for the qualitative part (not all items are relevant).

STROBE Statement—Checklist of items that should be included in reports of ***cross-sectional studies***

|  | Item No | Recommendation |  |
| --- | --- | --- | --- |
| **Title and abstract** | 1 | (*a*) Indicate the study’s design with a commonly used term in the title or the abstract | √ |
|  |  | (*b*) Provide in the abstract an informative and balanced summary of what was done and what was found | √ |
| Introduction | | |  |
| Background/rationale | 2 | Explain the scientific background and rationale for the investigation being reported | √ |
| Objectives | 3 | State specific objectives, including any prespecified hypotheses | √  We do state specific objectives |
| Methods | | |  |
| Study design | 4 | Present key elements of study design early in the paper | √  Study design is described. |
| Setting | 5 | Describe the setting, locations, and relevant dates, including periods of recruitment, exposure, follow-up, and data collection | √  Is described. |
| Participants | 6 | (*a*) Give the eligibility criteria, and the sources and methods of selection of participants | √  Is described |
| Variables | 7 | Clearly define all outcomes, exposures, predictors, potential confounders, and effect modifiers.  Give diagnostic criteria, if applicable | √  Outcomes and themes (views of nursing staff of implementation and feasibility SNG) are clearly described |
| Data sources/ measurement | 8* | For each variable of interest, give sources of data and details of methods of assessment (measurement). Describe comparability of assessment methods if there is more than one group | *√*  *Is described* |
| Bias | 9 | Describe any efforts to address potential sources of bias | √  Is described |
| Study size | 10 | Explain how the study size was arrived at | √  Is described |
| Quantitative variables | 11 | Explain how quantitative variables were handled in the analyses. If applicable, describe which groupings were chosen and why | √  Is described |
| Statistical methods | 12 | (*a*) Describe all statistical methods, including those used to control for confounding |  |
|  |  | (*b*) Describe any methods used to examine subgroups and interactions | Not relevant |
|  |  | (*c*) Explain how missing data were addressed | √  Is described |
|  |  | (*d*) If applicable, describe analytical methods taking account of sampling strategy | Not applicable |
|  |  | (*e*) Describe any sensitivity analyses | Not relevant |
| Results | | |  |
| Participants | 13* | (a) Report numbers of individuals at each stage of study—eg numbers potentially eligible, examined for eligibility, confirmed eligible, included in the study, completing follow-up, and analysed | √  Is described |
|  |  | (b) Give reasons for non-participation at each stage |  |
|  |  | (c) Consider use of a flow diagram | √  Is used. |
| Descriptive data | 14* | (a) Give characteristics of study participants (eg demographic, clinical, social) and information on exposures and potential confounders | √  Is described |
|  |  | (b) Indicate number of participants with missing data for each variable of interest | √  Is described |
| Outcome data | 15* | Report numbers of outcome events or summary measures | √  Is described |
| Main results | 16 | (*a*) Give unadjusted estimates and, if applicable, confounder-adjusted estimates and their precision (eg, 95% confidence interval). Make clear which confounders were adjusted for and why they were included | Not applicable |
|  |  | (*b*) Report category boundaries when continuous variables were categorized | Not applicable |
|  |  | (*c*) If relevant, consider translating estimates of relative risk into absolute risk for a meaningful time period | Not applicable |
| Other analyses | 17 | Report other analyses done—eg analyses of subgroups and interactions, and sensitivity analyses | Not applicable |
| Discussion | | |  |
| Key results | 18 | Summarise key results with reference to study objectives | √  Is described |
| Limitations | 19 | Discuss limitations of the study, taking into account sources of potential bias or imprecision. Discuss both direction and magnitude of any potential bias | √  Is described |
| Interpretation | 20 | Give a cautious overall interpretation of results considering objectives, limitations, multiplicity of analyses, results from similar studies, and other relevant evidence | √  Is described |
| Generalisability | 21 | Discuss the generalisability (external validity) of the study results | √  Is described |
| Other information | | |  |
| Funding | 22 | Give the source of funding and the role of the funders for the present study and, if applicable, for the original study on which the present article is based | √  Is described |

*Give information separately for exposed and unexposed groups.

**Note:** An Explanation and Elaboration article discusses each checklist item and gives methodological background and published examples of transparent reporting. The STROBE checklist is best used in conjunction with this article (freely available on the Web sites of PLoS Medicine at http://www.plosmedicine.org/, Annals of Internal Medicine at http://www.annals.org/, and Epidemiology at http://www.epidem.com/). Information on the STROBE Initiative is available at [www.strobe-statement.org](http://www.strobe-statement.org/).

**Feasibility and Implementation of the Stroke Nursing Guideline in the care of patients with stroke: A Mixed Methods Study**

This study concerns mixed methods study.

We used the Strobe statement for the reporting of the quantitative part of the study and the COREQ for the qualitative part (not all items are relevant).

**Consolidated criteria for reporting qualitative studies (COREQ): 32-item checklist**

Developed from:

Tong A, Sainsbury P, Craig J. Consolidated criteria for reporting qualitative research (COREQ): a 32-item checklist for interviews and focus groups. *International Journal for Quality in Health Care*. 2007. Volume 19, Number 6: pp. 349 – 357

**YOU MUST PROVIDE A RESPONSE FOR ALL ITEMS. ENTER N/A IF NOT APPLICABLE**

| **No. Item** | **Guide questions/description** | **Reported on Page #** |
| --- | --- | --- |
| **Domain 1: Research team and reﬂexivity** |  |  |
| *Personal Characteristics* |  |  |
| 1. Inter viewer/facilitator | Which author/s conducted the inter view or focus group? | Results  13 |
| 2. Credentials | What were the researcher’s credentials? E.g. PhD, MD | Methods  13 |
| 3. Occupation | What was their occupation at the time of the study? | Methods  13 |
| 4. Gender | Was the researcher male or female? | N/A |
| 5. Experience and training | What experience or training did the researcher have? | Methods  13 |
| *Relationship with participants* |  |  |
| 6. Relationship established | Was a relationship established prior to study commencement? | Yes |
| 7. Participant knowledge of the interviewer | What did the participants know about the researcher? e.g. personal goals, reasons for doing the research | Yes – participants were informed beforehand. |
| 8. Interviewer characteristics | What characteristics were reported about the inter viewer/facilitator? e.g. Bias, assumptions, reasons and interests in the research topic | Methods  Yes |
| **Domain 2: study design** |  |  |
| *Theoretical framework* |  |  |
| 9. Methodological orientation and Theory | What methodological orientation was stated to underpin the study? e.g. grounded theory, discourse analysis, ethnography, phenomenology, content analysis | Methods  Content analysis  (no other theoretical underpinnings) |
| *Participant selection* |  |  |
| 10. Sampling | How were participants selected? e.g. purposive, convenience, consecutive, snowball | Methods  Purposive. |
| 11. Method of approach | How were participants approached? e.g. face-to-face was used. | Methods  Face to face pg. 13 |
| 12. Sample size | How many participants were in the study?  In the qualtitative part, 16 nurses and auxiliary nurses (N=8 each group, respectively) took part in three focus group interviews. | Results  See pg. 10 and 16. |
| 13. Non-participation | How many people refused to participate or dropped out? Reasons? | Methods  Not reported |
| *Setting* |  |  |
| 14. Setting of data collection | Where was the data collected? e.g. home, clinic, workplace | Methods  Is described on pg. 15 |
| 15. Presence of non-participants | Was anyone else present besides the participants and researchers? | Results  No |
| 16. Description of sample | What are the important characteristics of the sample? e.g. demographic data, date | Results  See pg. 16 and table 3. |
| *Data collection* |  |  |
| 17. Interview guide | Were questions, prompts, guides provided by the authors? Was it pilot tested? | Methods  See table 1 |
| 18. Repeat interviews | Were repeat inter views carried out? If yes, how many? | N/A  3 interviews were carried out. |
| 19. Audio/visual recording | Did the research use audio or visual recording to collect the data? | Methods  Yes audio recordings. Pg. 13 |
| 20. Field notes | Were ﬁeld notes made during and/or after the inter view or focus group? | Methods  Yes pg. 13 |
| 21. Duration | What was the duration of the inter views or focus group? | Methods |
| 22. Data saturation | Was data saturation discussed? | Methods |
| 23. Transcripts returned | Were transcripts returned to participants for comment and/or correction? | N/A  Summaries of transcripts were made for participants which they could verify. |
| **Domain 3: analysis and ﬁndings** |  |  |
| *Data analysis* |  |  |
| 24. Number of data coders | How many data coders coded the data? | Methods  3 authors took part in the analysis |
| 25. Description of the coding tree | Did authors provide a description of the coding tree? | NA |
| 26. Derivation of themes | Were themes identiﬁed in advance or derived from the data? | Methods  Themes were derived from the data. |
| 27. Software | What software, if applicable, was used to manage the data? | NVivo  NA |
| 28. Participant checking | Did participants provide feedback on the ﬁndings? | Strengths and limitations  Yes |
| *Reporting* |  |  |
| 29. Quotations presented | Were participant quotations presented to illustrate the themes/ﬁndings? Was each quotation identiﬁed? e.g. participant number | Results  Yes – see table 8 |
| 30. Data and ﬁndings consistent | Was there consistency between the data presented and the ﬁndings? | Relationship to existing knowledge  YES |
| 31. Clarity of major themes | Were major themes clearly presented in the ﬁndings? | Results  Themes supported the quantitative findings. |
| 32. Clarity of minor themes | Is there a description of diverse cases or discussion of minor themes? | Discussion  No relevant in this study. |

**Once you have completed this checklist, please save a copy and upload it as part of your submission. When requested to do so as part of the upload process, please select the file type: *Checklist*. You will NOT be able to proceed with submission unless the checklist has been uploaded. Please DO NOT** **include this checklist as part of the main manuscript document. It must be uploaded as a separate file.**
