# Supplementary material for: Implementation and feasibility of the stroke nursing guideline in the care of patients with stroke: a mixed methods study
Source: BMC Nurs. 2017 Dec 1;16:72. doi: 10.1186/s12912-017-0262-y (PMC5709925; doi:10.1186/s12912-017-0262-y)
Supplement: Supplementary file 2 — Interview guide. (DOCX 16 kb) [file 12912_2017_262_MOESM2_ESM.docx]

**Interview Guide** (Additional file 1)

**________________________________________________________________________________________________________**

1. Has the implementation of the Stroke Nursing Guidelines:

- Increased your knowledge and/or changed nursing care of patients with stroke and their families and if so, how?

- How have the changes been on:

-Activities of daily living

-Mobility

-Emotional wellbeing, particularly depression

-Do you use PHQ-9 to assess symptoms of depression

-Patient teaching

-Discharge planning

-Pain

-Falls

- What is helpful and what is less helpful about the guidelines?

- What is helpful to teach students and new staff?

- What needs to be improved to make the guidelines more useful?

- If these guidelines are/were not used what should be used instead?

- What needs to be done differently regarding the process of implementing the guidelines, particularly regarding activities of daily living, mobility, depression, patient teaching, discharge planning, pain and falls?

2. On a scale from 1-10, how active have you been in the implementation process?

3. On a scale from 1-10, how useful have the guidelines been, i.e. do you consider the guidelines having improved the nursing care for this patient group?

____________________________________________________________________________________________________________
